# Intravoxel incoherent motion MRI for early detection and assessment of renal injury after cardiac arrest and resuscitation in a rat model

**DOI:** 10.1186/s41747-026-00729-8

**Published:** 2026-05-13

**Authors:** Jie Zhang, Na Zhan, Haoyi Ye, Siqi Liu, Yunke Tan, Jinzhao Zhang, Teng Huang, Zhihua Wu, Zhengfei Yang, Zhifeng Liu

**Affiliations:** 1 Department of Emergency Medicine, Sun Yat-sen Memorial Hospital, Sun Yat-sen University, Guangzhou, China; 2 Key Laboratory of Protein Modification and Degradation, School of Basic Medical Sciences, Guangzhou Medical University, Guangzhou, China; 3 Department of Radiology, The Fourth Affiliated Hospital of Guangzhou Medical University, Guangzhou, China; 4 Department of Intensive Care Medicine, Sun Yat-sen Memorial Hospital, Sun Yat-sen University, Guangzhou, China

**Keywords:** Cardiopulmonary resuscitation, Kidney, Magnetic resonance imaging, Post-cardiac arrest syndrome, Tight junction proteins

## Abstract

**Objective:**

To investigate the utility of intravoxel incoherent motion (IVIM) magnetic resonance imaging (MRI) for early detection of renal injury 24 h after cardiac arrest and cardiopulmonary resuscitation.

**Materials and methods:**

Twenty-four male Sprague–Dawley rats were randomized into cardiac arrest (*n* = 15) and sham-operated (*n* = 9) groups. Five cardiac arrest rats died during the procedure, resulting in 10 cardiac arrest and 9 sham rats for analysis. IVIM-MRI and renal parameters were assessed 24 h after cardiac arrest and cardiopulmonary resuscitation. Diffusion-related parameters, including apparent diffusion coefficient (ADC), true diffusion coefficient (D), pseudo-diffusion coefficient (D*), perfusion fraction (f), and exponential ADC (eADC), were obtained. The primary imaging endpoint was defined as D. Following completion of MRI, renal function, histopathology, and expression of apoptosis markers (Bcl-2, Caspase-3), water channel protein aquaporin-3, and tight junction proteins (zonula occludens-1, occludin) were subsequently evaluated.

**Results:**

Despite preserved conventional renal function, IVIM MRI detected early renal alterations 24 h after cardiac arrest. Compared with the sham group, eADC, D, ADC, D*, and f values were all significantly reduced. Histopathological analysis revealed pronounced tubular epithelial injury, increased apoptosis (Bcl-2, *p* < 0.001; Caspase-3, *p* = 0.010), and reduced expression of aquaporin-3 (*p* = 0.039), zonula occludens-1 (*p* < 0.001), and occludin (*p* < 0.001).

**Conclusion:**

IVIM MRI enables early, noninvasive detection of renal injury 24 h after cardiac arrest and CPR, reflecting tubular injury, tight junction disruption, and perfusion deficits, potentially facilitating timely intervention and improving prognosis.

**Relevance statement:**

This approach may facilitate individualized therapy and improve post-cardiac arrest syndrome outcome.

**Key Points:**

- The long-term outcomes of cardiac arrest remain poor due to post-cardiac arrest syndrome.
- IVIM MRI enables early, noninvasive detection of renal injury after cardiac arrest and cardiopulmonary resuscitation.
- This approach may facilitate individualized therapy and improve post-cardiac arrest syndrome.

**Graphical Abstract:**

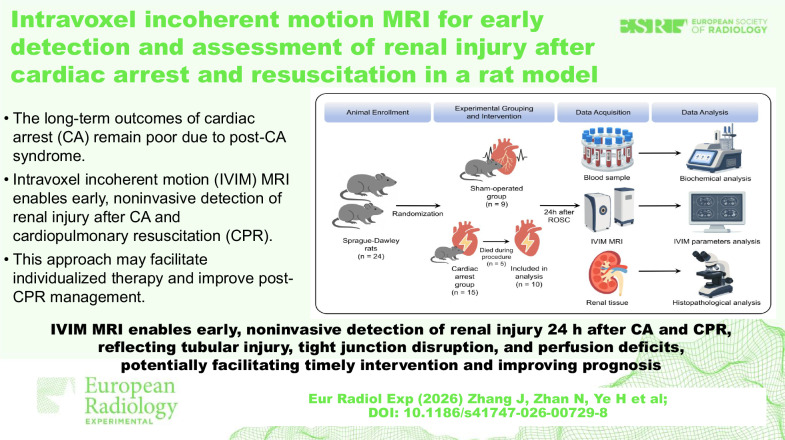

## Background

Despite advances in cardiopulmonary resuscitation (CPR) improving short-term survival after cardiac arrest (CA) [1], long-term outcomes remain poor, mainly due to post-cardiac arrest syndrome (PCAS). PCAS is a complex condition involving myocardial dysfunction, neurological impairment, systemic ischemia-reperfusion injury (IRI), inflammation, and multi-organ damage, with acute kidney injury (AKI) being particularly common and clinically important [2]. AKI affects about 30% of in-hospital and up to 48.3% of out-of-hospital CA survivors, correlating with higher mortality, prolonged hospitalization, and increased need for renal replacement therapy [3, 4]. Survivors often face persistent renal dysfunction and considerable healthcare burdens [5].

To better investigate this complex injury, animal models that replicate systemic ischemia–reperfusion after CA are essential. CA followed by return of spontaneous circulation (ROSC) causes systemic IRI affecting multiple organs, including the kidneys, heart, and brain [6, 7]. Unlike traditional AKI models focusing on localized renal ischemia, the CA-ROSC model better mimics clinically relevant systemic IRI with global hypoxia and reperfusion injury [4, 8]. Preclinical CA-ROSC models reproduce clinical AKI features such as elevated serum creatinine (Cr), blood urea nitrogen (BUN), tubular swelling, and inflammatory infiltration, reflecting systemic IRI beyond localized renal ischemia [8]. This complex injury involves inflammation, oxidative stress, and microcirculatory dysfunction, not fully captured in isolated ischemia models [9]. Thus, CA-ROSC models offer a valuable framework to study molecular mechanisms and diagnostic challenges of multi-organ damage after CA [10].

Despite these advances in modeling, early diagnosis of AKI remains challenging due to limitations in conventional biomarkers. Early diagnosis of AKI at a stage earlier than overt AKI is challenging, since conventional biomarkers such as serum Cr and urine output lack sensitivity in the early phase and only rise after significant nephron loss [11, 12]. Serum Cr mainly reflects glomerular filtration changes and may stay normal despite ongoing tubular and microvascular injury. Renal biopsy, the diagnostic gold standard, is invasive and unsuitable for routine use in critically ill patients [11]. Therefore, noninvasive and reliable early detection methods are urgently needed.

Early renal injury after CA activates multiple cell death pathways, including apoptosis, ferroptosis, and autophagy, resulting in tubular epithelial cell loss and functional decline [13, 14]. This is accompanied by disruption of adhesion and tight junction proteins such as occludin and zonula occludens-1 (ZO-1), with ultrastructural changes observable *via* electron microscopy [15]. Impairment of tight junctions increases paracellular permeability, promoting tubular backleak and interstitial edema, thereby disturbing intrarenal water homeostasis. Alterations in aquaporin-3, a basolateral water channel essential for water transport, have also been documented in acute ischemic injuries, serving as an early marker of tubular dysfunction [16].

Intravoxel incoherent motion (IVIM) magnetic resonance imaging (MRI), a multi-*b*-value diffusion-weighted technique originally proposed by Dixon to separate diffusion and perfusion signals noninvasively [8, 17], enables simultaneous assessment of tissue microstructure and microvascular perfusion. IVIM-derived parameters—including apparent diffusion coefficient (ADC), true diffusion coefficient (D), and pseudo-diffusion coefficient (D*)—have been successfully applied to detect subclinical renal dysfunction, predict delayed graft function, and diagnose early diabetic nephropathy [18–20]. These parameters reflect alterations in renal pathophysiology, supporting their utility as early, noninvasive biomarkers for AKI after CA.

At the cellular and tissue levels, IRI disrupts cellular energy metabolism, cytoskeletal integrity, and tight junction proteins (occludin, claudins, ZO-1), leading to loss of epithelial polarity and barrier dysfunction [21, 22]. These microstructural alterations, including tubular swelling, cytoskeletal disorganization, and tight junction breakdown, increase paracellular permeability and restrict true molecular diffusion, as evidenced by decreases in D and ADC values [23]. Concurrently, microvascular injury—characterized by endothelial damage and capillary rarefaction—impairs renal perfusion, reflected in alterations of perfusion-sensitive IVIM parameters such as D* and f [24]. Although IVIM MRI has shown promise in detecting renal ischemia, existing studies have predominantly focused on localized ischemia models [17, 25]. In contrast, systemic IRI following CA-ROSC presents a more complex clinical condition characterized by multi-organ dysfunction, including AKI, which remains insufficiently investigated using IVIM techniques. Furthermore, the association between IVIM-derived parameters and molecular markers such as aquaporin-3—a key regulator of renal water transport and injury response—has yet to be fully clarified. This study seeks to address these gaps by integrating IVIM MRI with aquaporin-3 expression analysis in a clinically relevant CA-ROSC rat model, aiming to enhance early detection and provide a deeper mechanistic understanding of renal injury following CA.

Based on previous studies [26, 27], we hypothesized that IVIM-derived diffusion and perfusion parameters at 24 h after ROSC would differ between the CA and sham groups and correlate with established markers of renal injury. Accordingly, this study aimed to systematically assess the potential of IVIM MRI as a sensitive, noninvasive imaging modality for the early detection of CA-induced renal IRI.

## Methods

Male Sprague–Dawley rats (250–300 g) were obtained from the Experimental Animal Center of Sun Yat-sen University (Guangzhou, China). Animals were maintained under specific pathogen-free conditions at 20–22 °C with a 12-h light/dark cycle and had free access to standard chow and water. Throughout the study, animals were monitored daily for general health status, body weight, grooming behavior, and signs of distress. The detailed procedures of the CA model, experimental procedures and statistical analysis are described in Supplementary Methods.

All experimental procedures were approved by the Institutional Animal Care and Use Committee of Sun Yat-sen Memorial Hospital, Sun Yat-sen University (Approval No. AP20240293), and conducted in accordance with the NIH Guide for the Care and Use of Laboratory Animals. Every effort was made to minimize animal suffering. Sodium pentobarbital was obtained from Sigma-Aldrich.

## Results

### Baseline and post-resuscitation physiological parameters and renal function markers

**Fig. 1:**
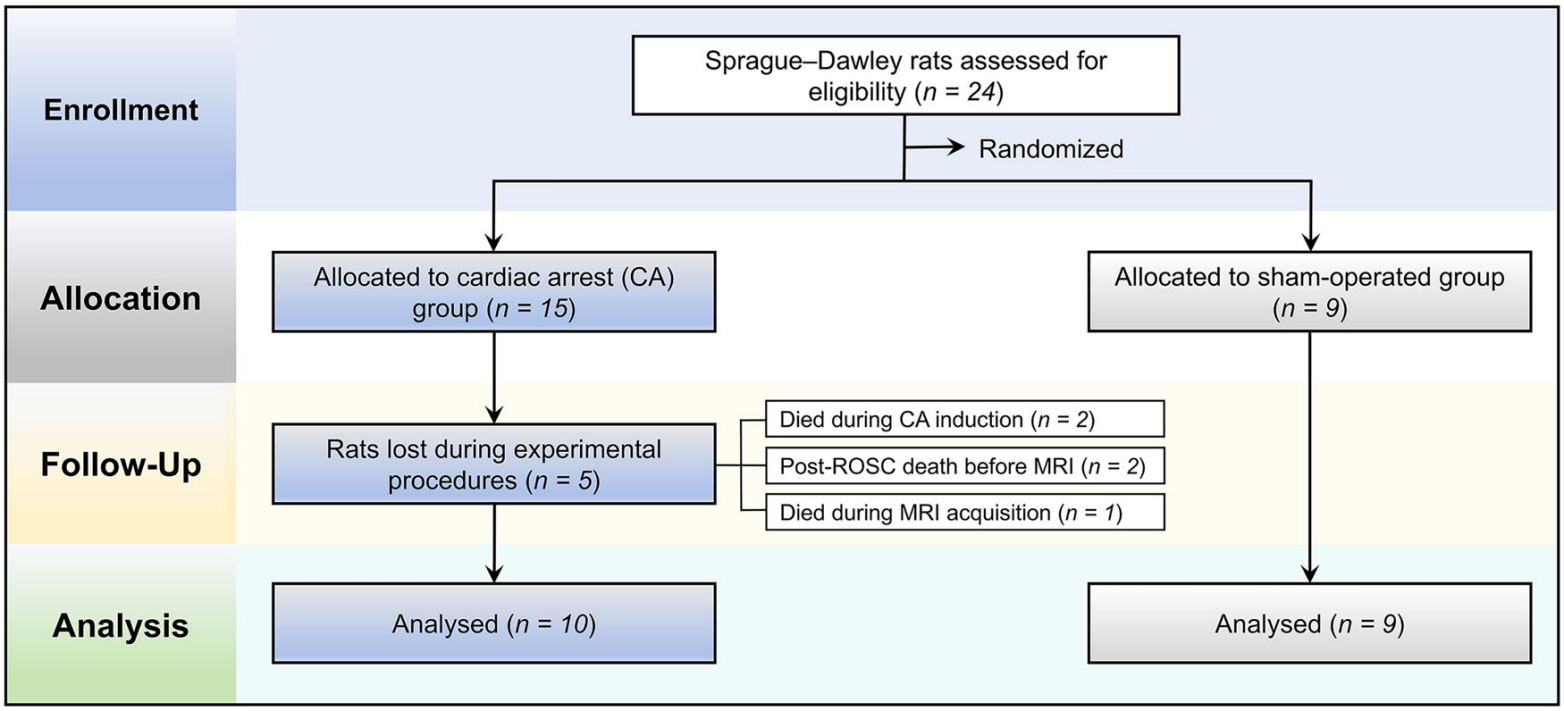
CONSORT-style flow diagram of animal randomization and experimental procedures. A total of 24 Sprague–Dawley rats were enrolled and randomly allocated to the cardiac arrest (CA) group or the sham-operated group. Five animals in the CA group were lost during the experimental procedures, including deaths occurring during CA induction (*n* = 2), after return of spontaneous circulation but prior to MRI acquisition (*n* = 2), and during MRI scanning (*n* = 1); all animals in the sham group survived. Consequently, 10 CA rats and 9 sham rats were included in the final analysis. At 24 h after return of spontaneous circulation (ROSC), blood samples were collected, intravoxel incoherent motion MRI was performed, and renal tissues were harvested for biochemical assays, histopathological evaluation, and Western blot analysis. CA, Cardiac arrest; MRI, Magnetic resonance imaging; ROSC, Return of spontaneous circulation

Of the 24 male Sprague–Dawley rats initially randomized, 15 were assigned to the CA group and 9 to the sham-operated group. In the CA group, five animals died during the experimental course: two during CA induction, two after successful resuscitation but prior to MRI acquisition, and one during MRI scanning. Thus, 10 CA rats achieved ROSC and completed IVIM-MRI and subsequent analyses. All sham-operated animals survived and were successfully imaged. Accordingly, the final analyses included 10 rats in the CA group and 9 rats in the sham group. The overall experimental design and study flow are summarized in a CONSORT-style flow diagram (Fig. 1). Baseline physiological and renal function parameters—including mean arterial pressure, heart rate, respiratory rate, peripheral oxygen saturation‒SpO₂, body temperature, Cr, and BUN—were comparable between groups, with no significant differences observed. Additionally, within each group, no significant changes were detected between pre- and post-resuscitation measurements. At 24 h after resuscitation, comparisons between groups also showed no significant differences in these parameters (Table 1). Plasma Cr and BUN levels measured before and 24 h after the procedure showed no significant within-group changes (ΔCr and ΔBUN, Table 1). Baseline physiological parameters, including anesthesia duration, were carefully monitored. The anesthesia time was separated into two phases: the CA model induction anesthesia and the IVIM MRI scanning anesthesia. The CA model anesthesia time was 60.3 ± 1.6 min in the CA group and 60.1 ± 1.2 min in the sham group, with no statistically significant difference (*p* = 0.730). The IVIM MRI anesthesia time was 7.06 ± 0.20 min for CA rats and 6.99 ± 0.19 min for sham rats, also without significant difference (*p* = 0.540). These comparable anesthesia durations between groups help exclude anesthesia time as a confounding factor influencing renal perfusion and diffusion parameters. These findings demonstrate that conventional biochemical markers did not detect renal injury at this early stage, while the observed decreases in IVIM perfusion-related parameters are unlikely to result from systemic hemodynamic instability, instead reflecting localized renal microcirculatory impairment.

**Table 1:** Combined baseline physiological parameters and postoperative renal function parameters in sham and cardiac arrest groups

Parameter	Group	Baseline (mean ± SD)	Post-cardiac arrest (mean ± SD)	Within-group *p*-value (baseline *vs*. post)	Between-groups *p*-value (baseline)	Between-groups *p*-value (post)
Body weight (g)	Cardiac arrest	254.1 ± 5.3	254.7 ± 6.3	0.616	0.654	0.601
	Sham	252.5 ± 6.1	253.3 ± 5.0	0.772		
Mean arterial pressure (mmHg)	Cardiac arrest	92.8 ± 4.5	89.1 ± 3.0	0.053	0.768	0.243
	Sham	92.0 ± 5.7	87.4 ± 4.4	0.075		
Body temperature (°C)	Cardiac arrest	37.0 ± 0.2	37.1 ± 0.2	0.678	0.068	0.347
	Sham	37.1 ± 0.2	37.0 ± 0.2	0.417		
Heart rate (beats/min)	Cardiac arrest	323.4 ± 6.6	329.5 ± 5.5	0.050	0.760	0.360
	Sham	323.3 ± 4.6	327.2 ± 4.9	0.105		
Respiratory rate (breaths/min)	Cardiac arrest	64.9 ± 1.8	67 ± 5.5	0.241	0.727	0.776
	Sham	64.8 ± 2.8	67.7 ± 4.5	0.121		
Oxygen saturation (SpO₂, %)	Cardiac arrest	97.3 ± 1.1	96.3 ± 1.6	0.052	0.110	0.812
	Sham	96.6 ± 1.3	96.1 ± 1.8	0.565		
Creatinine (µmol/L)	Cardiac arrest	66.4 ± 3.1	66.5 ± 4.3	0.871	0.068	0.092
	Sham	64.0 ± 2.7	63.0 ± 4.0	0.559		
Blood urea nitrogen (mmol/L)	Cardiac arrest	6.8 ± 1.2	8.0 ± 2.1	0.073	0.255	0.239
	Sham	6.7 ± 1.6	7.0 ± 1.1	0.613		
Δcreatinine (µmol/L)	Cardiac arrest		-0.1 ± 2.1			0.335
	Sham		1.0 ± 2.7			
ΔBlood urea nitrogen (mmol/L)	Cardiac arrest		-1.3 ± 2.1			0.691
	Sham		-0.9 ± 2.3			

Values are presented as mean ± SD. Baseline physiological variables were measured prior to intervention, and renal function indices were assessed before cardiac arrest and 24 h after cardiopulmonary resuscitation. Δ represents the change from baseline. Statistical comparisons between groups revealed no significant differences

*SD* Standard deviation

### Histopathology and ultrastructural alterations 24 h post-ROSC

**Fig. 2:**
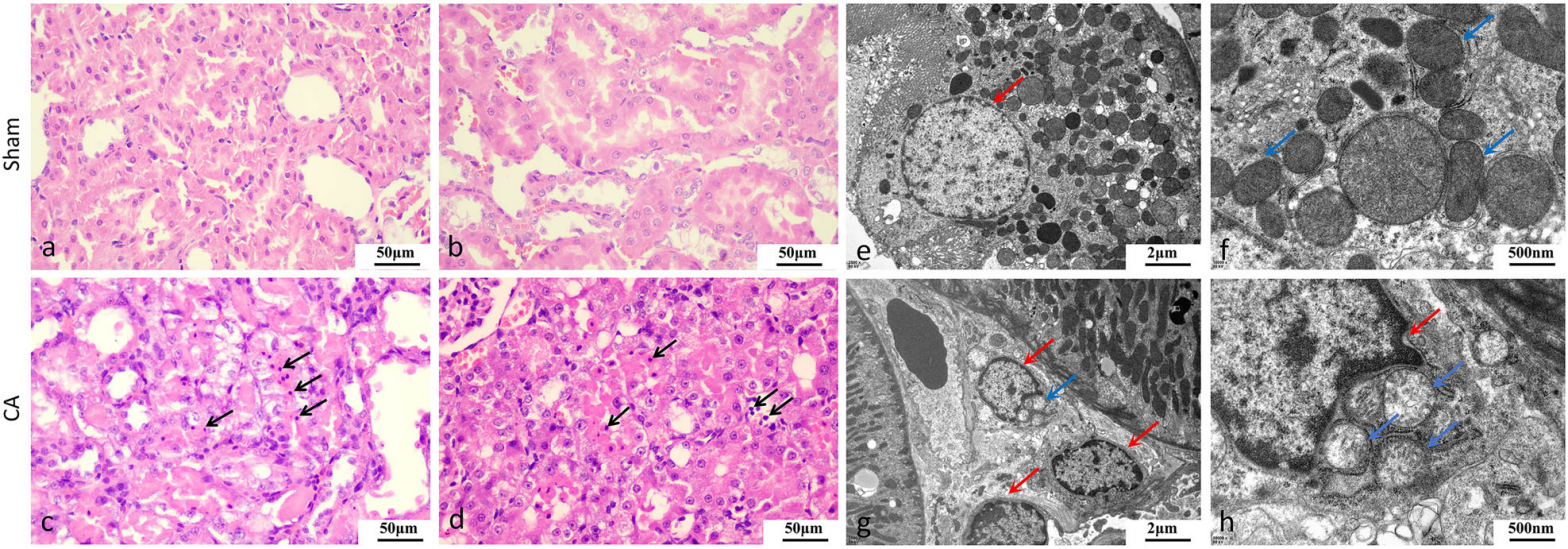
Histological and ultrastructural features of renal tubular injury. Hematoxylin and eosin (H&E) staining of kidney tissues showed normal tubular morphology in sham kidneys (**a**, **b**) and pronounced tubular injury in CA kidneys (**c**, **d**). Black arrows indicate areas of cytoplasmic eosinophilia and nuclear pyknosis. Partial epithelial detachment and flattening are also visible in CA kidneys. Scale bars: 50 μm (**a**–**d**). TEM analysis revealed nuclear and mitochondrial ultrastructure. Sham kidneys (**e**, **f**) exhibited normal nuclei and mitochondria. CA kidneys (**g**, **h**) showed chromatin condensation, nuclear shrinkage, and mitochondrial cristae loss or fragmentation. Red arrows indicate nuclei; blue arrows indicate mitochondria. Scale bars: 2 μm (**e**, **g**), 0.5 μm (**f**, **h**). CA, Cardiac arrest; TEM, Transmission electron microscopy

Hematoxylin and eosin staining revealed tubular injury in CA kidneys, characterized by increased cytoplasmic eosinophilia, nuclear pyknosis, and partial epithelial detachment and flattening, whereas sham kidneys displayed normal morphology (Fig. 2a–d). Transmission electron microscopy confirmed ultrastructural abnormalities in CA kidneys, including nuclear shrinkage, chromatin condensation, compromised nuclear membrane integrity, and mitochondrial reduction with cristae loss or fragmentation (Fig. 2g, h). Sham kidneys showed normal nuclear and mitochondrial ultrastructure (Fig. 2e, f). Renal histopathological injury scores, semi-quantitatively assessed on H&E-stained sections, were significantly elevated in the CA group (mean ± standard deviation: 1.78 ± 0.44) compared to the sham group (0.44 ± 0.53; *p* < 0.001), indicating pronounced apoptosis and cellular damage following cardiac arrest. Similarly, ultrastructural injury scores evaluated *via* transmission electron microscopy were markedly increased in the CA group (1.67 ± 0.44) relative to shams (0.22 ± 0.44; *p* < 0.001), reflecting severe mitochondrial and nuclear ultrastructural damage. These assessments were independently performed by two blinded observers, with high interobserver reliability confirmed by intraclass correlation coefficients.

### Tight junctions and tubular injury markers

**Fig. 3:**
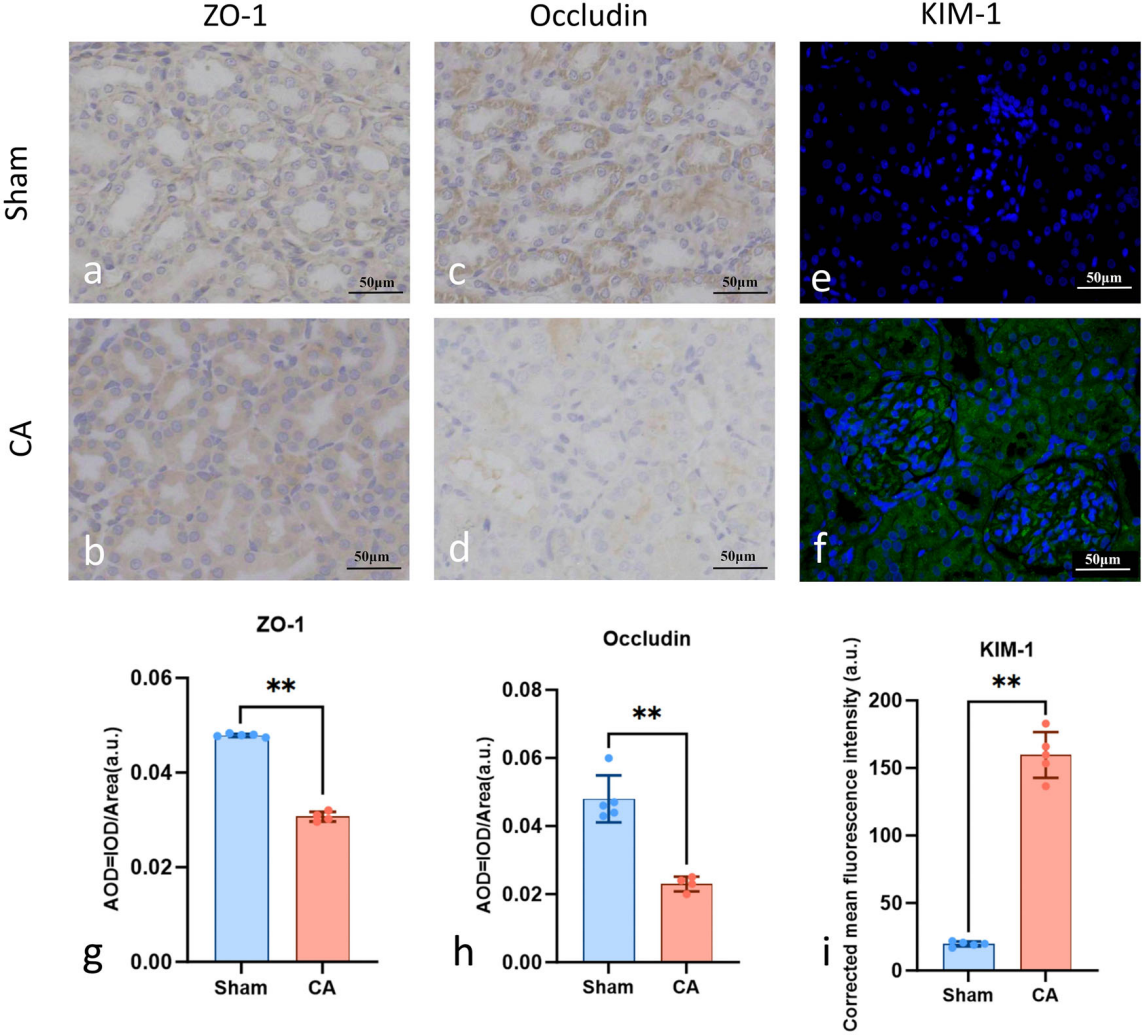
Detection of tight junction proteins and renal injury by immunohistochemistry and immunofluorescence. Immunohistochemistry demonstrated reduced ZO-1 and occludin expression in CA kidneys compared with sham controls (**a**–**d**, **g**, **h**). Immunofluorescence revealed increased KIM-1 signals in the tubular epithelium of CA kidneys (**e**, **f**, **i**). Semi-quantitative analysis confirmed these changes. Data are presented as mean ± standard deviation; the *y*-axis in **i** is abbreviated as MFI (a.u.) for clarity. IOD represents cumulative staining intensity used for semi-quantitative evaluation. Scale bars: 50 μm. Asterisks indicate statistical significance (** *p* < 0.001). AOD, Average optical density; CA, Cardiac arrest; IOD, Integrated optical density; KIM-1, Kidney injury molecule‑1; MFI, Mean fluorescence intensity; ZO-1, Zonula occludens-1

Immunohistochemistry and immunofluorescence analyses demonstrated a pronounced disruption of tubular epithelial tight junctions in CA kidneys. In contrast to the strong, continuous membranous staining observed in sham animals, ZO-1 and occludin in the CA group exhibited markedly attenuated and fragmented junctional patterns (Fig. 3a–d). Quantitative assessments confirmed significant reductions in ZO-1 (average optical density: 0.031 ± 0.001 *versus* 0.048 ± 0.001, *p* < 0.001) and occludin expression (0.023 ± 0.002 *versus* 0.048 ± 0.007, *p* < 0.001; Fig. 3g, h). Furthermore, kidney injury molecule‑1 fluorescence intensity was substantially elevated in CA kidneys compared with sham controls (159.68 ± 16.95 *versus* 19.73 ± 1.90, *p* < 0.001; Fig. 3e, f, i), indicating the presence of early and prominent tubular epithelial injury.

#### Apoptosis-related proteins

**Fig. 4:**
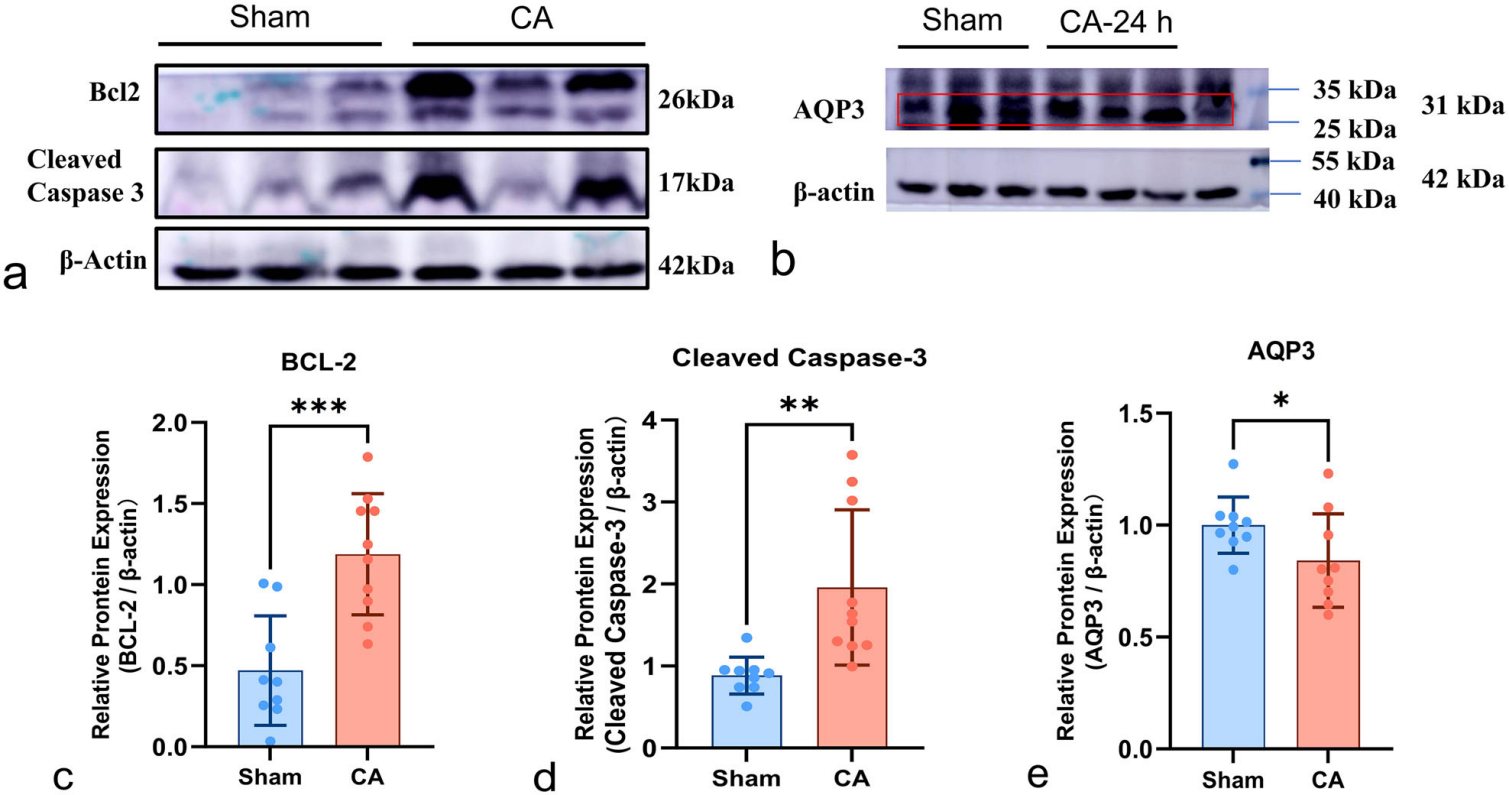
Expression of apoptosis-related proteins and AQP3 in cardiac arrest kidneys. Representative Western blot images show the expression of Bcl-2, cleaved caspase-3, AQP3, and β-actin in renal tissues from sham and cardiac arrest (CA) rats (**a**, **b**). In **a**, the upper band corresponds to Bcl-2. The molecular weight marker lane in **b** was removed for clarity. Quantitative analysis demonstrated significantly increased expression of Bcl-2 (**c**) and cleaved Caspase-3 (**d**), along with significantly decreased expression of AQP3 (**e**), in CA kidneys compared with sham controls. Protein levels were normalized to β-actin. Data are presented as mean ± standard deviation. Asterisks indicate statistical significance (* *p* < 0.05, ** *p* < 0.001). AQP3, Aquaporin-3; Bcl-2, B‑cell lymphoma-2 protein; CA, Cardiac arrest

Western blot analysis revealed a significant upregulation of Bcl-2 in CA kidneys (1.20 ± 0.41 *versus* 0.48 ± 0.35, *p* < 0.001, Fig. 4a, c), indicating a possible early cellular protective response. Meanwhile, cleaved caspase-3 levels were also significantly increased (1.24 ± 0.51 *versus* 0.66 ± 0.33, *p* = 0.01; Fig. 4a, d), reflecting activation of apoptotic execution pathways. These findings suggest a dynamic balance between pro-survival and pro-apoptotic signaling at 24 h after ROSC.

#### Aquaporin-3 expression

Western blot analysis revealed a significant downregulation of aquaporin-3 expression in the CA group relative to shams (0.84 ± 0.21 *versus* 1.00 ± 0.13, *p* = 0.039; Fig. 4b, e). The reduction in aquaporin-3 indicates impaired tubular water transport capacity and potential disruption of epithelial polarity in the early phase following CA.

#### IVIM MRI assessment of renal diffusion and perfusion

**Fig. 5:**
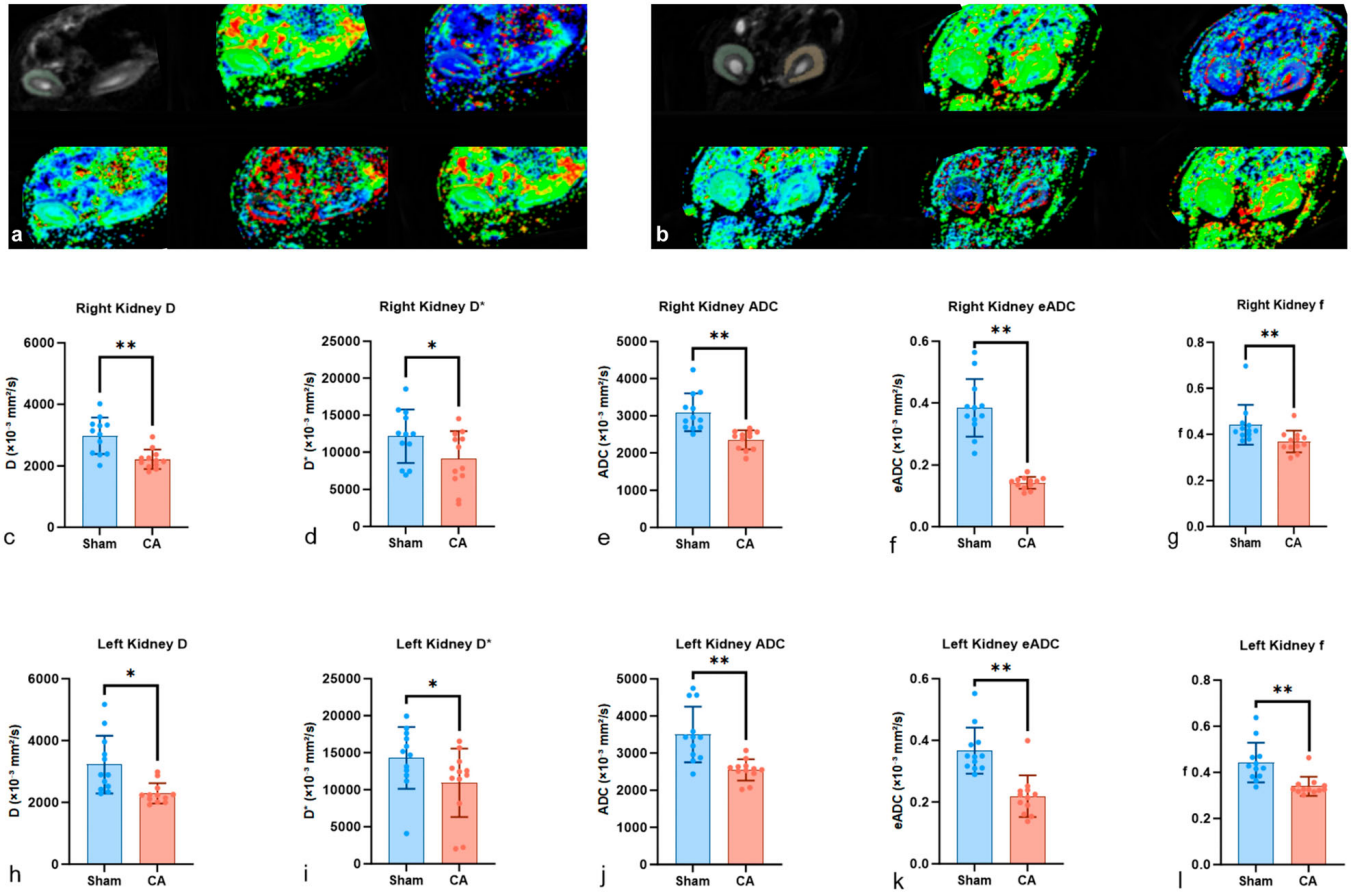
IVIM parameter maps and quantitative analysis of renal diffusion and perfusion in sham and CA rat kidneys. **a** Representative IVIM parametric maps from the sham group, including anatomical reference images (Source), diffusion coefficient (D), pseudo-diffusion coefficient (D*), apparent diffusion coefficient (ADC), effective apparent diffusion coefficient (eADC), and perfusion fraction (f), demonstrating a relatively homogeneous distribution across the renal parenchyma. **b** Representative IVIM parametric maps from the CA group at 24 h post-resuscitation, showing increased spatial heterogeneity with reduced diffusion- and perfusion-related parameters. **c**–**g** Quantitative whole-kidney ROI analysis of D, D*, ADC, eADC, and f in the right kidney. **h**–**l** Quantitative whole-kidney ROI analysis of D, D*, ADC, eADC, and f in the left kidney. Data are presented as mean ± standard deviation. Whole-kidney ROIs were delineated along the outer contour of the renal parenchyma without separation of the cortex and medulla. Asterisks indicate statistical significance (* *p* < 0.05; ** *p* < 0.001). IVIM, Intravoxel incoherent motion; ROI, Region of interest

IVIM-derived parametric maps revealed early alterations in renal diffusion and microvascular perfusion 24 h after CA (Fig. 5a, b). ROIs were manually delineated along the outer contour of the renal parenchyma without separating the cortex from the medulla. In sham-operated rats, parametric maps of ADC, D, D*, f, and eADC exhibited a relatively homogeneous distribution throughout the renal parenchyma, indicating preserved diffusion and perfusion characteristics (Fig. 5a). In contrast, kidneys from the CA group demonstrated pronounced spatial heterogeneity, characterized by diffusely reduced ADC and D values, accompanied by decreased D* and f, suggesting impaired water diffusion and microvascular perfusion at 24 h post-resuscitation (Fig. 5b). These visual alterations were consistent with the quantitative IVIM parameter changes observed in subsequent analyses and correspond well with known pathophysiological changes following renal IRI.

Quantitative evaluation confirmed significant decreases across all diffusion- and perfusion-related metrics. In the right kidney, D declined from 3,036 ± 591 to 2,237 ± 336 × 10⁻⁶ mm²/s (*p* = 0.002), D* from 11,612 ± 3,882 to 7,799 ± 3,128 × 10⁻⁶ mm²/s (*p* = 0.030), ADC from 3,361 ± 516 to 2,380 ± 264 × 10⁻⁶ mm²/s (*p* < 0.001), f from 0.39 ± 0.09 to 0.14 ± 0.05 × 10⁻⁶ mm²/s (*p* < 0.001), and eADC from 0.46 ± 0.09 to 0.36 ± 0.05 × 10⁻⁶ mm²/s (*p* = 0.010) (Fig. 5c–g). Similar patterns were observed in the left kidney, with D declining from 3,303 ± 1,050 to 2,310 ± 361 × 10⁻⁶ mm²/s (*p* = 0.012), D* from 14,558 ± 4,844 to 9,887 ± 4,722 × 10⁻⁶ mm²/s (*p* = 0.048), ADC from 3,602 ± 739 to 2,545 ± 314 × 10⁻⁶ mm²/s (*p* = 0.001), f from 0.38 ± 0.08 to 0.23 ± 0.06 ×10⁻⁶ mm²/s (*p* < 0.001), and eADC from 0.47 ± 0.09 to 0.34 ± 0.05 × 10⁻⁶ mm²/s (*p* = 0.001) (Fig. 5h–l).

Intraclass correlation coefficients for ADC, D, D*, f, and eADC indicated good-to-excellent reproducibility of ROI-based measurements in both CA and sham groups. Specifically, intraclass correlation coefficients ranged from 0.831 to 0.961 in the CA group and from 0.892 to 0.992 in the sham group across both kidneys, exceeding the accepted reliability threshold (intraclass correlation coefficient > 0.75) and confirming the robustness of the imaging protocol.

Further analysis using averaged values from both kidneys reinforced these findings. Compared with sham controls, CA animals exhibited significantly reduced D values (2,273.20 ± 274.99 *versus* 3,169.29 ± 710.93 × 10⁻⁶ mm²/s, *p* = 0.020), as well as significant decreases in D* (8,843.19 ± 351.76 *versus* 13,084.80 ± 3,833.66 × 10⁻⁶ mm²/s, *p* = 0.023), ADC (2,462.30 ± 258.80 *versus* 3,460.29 ± 453.39 × 10⁻⁶ mm²/s, *p* < 0.001), perfusion fraction f (0.19 ± 0.04 *versus* 0.37 ± 0.07, *p* < 0.001), and eADC (0.35 ± 0.05 *versus* 0.47 ± 0.08 × 10⁻³ mm²/s, *p* = 0.001). All statistical analyses were performed at the individual animal level, as specified in the “Methods.”

These findings indicate that IVIM MRI detects microstructural and functional alterations in the kidney following CA, even in cases where biochemical markers remain unchanged.

## Discussion

In this study, we employed a clinically relevant CA-ROSC rat model that closely replicates the systemic IRI observed in human patients. This robust biological rationale and well-established model provide a strong foundation for exploring early mechanisms of renal injury and potential diagnostic methods. Using IVIM MRI, we observed evidence of early renal IRI at 24 h post-ROSC—an interval during which conventional renal biomarkers, such as Cr and BUN, remained within normal ranges. In the CA group, IVIM-derived parameters, including ADC, D, D*, f, and eADC, showed marked reductions, reflecting restricted water diffusion caused by cellular edema and impaired microcirculation. While similar IVIM alterations have been reported in localized AKI models, their application in CA-induced systemic IRI remains limited. In our study, these imaging findings correlated with early tubular injury markers, including apoptotic signaling activation, disruption of tight junction proteins (ZO-1, occludin), and altered aquaporin-3 expression. Notably, the imaging changes preceded elevations in plasma Cr and BUN, underscoring IVIM MRI as a sensitive, noninvasive modality for early detection of renal injury. Our focus on early injury within 24 h post-CA reflects subclinical ischemia–reperfusion damage, a stage often undetectable by conventional biomarkers. By facilitating earlier detection, IVIM MRI offers a crucial window for timely intervention that may ultimately improve patient outcomes.

Our findings align with and extend previous evidence demonstrating the sensitivity of IVIM parameters to microstructural and functional renal alterations across diverse injury models. Building on the early detection highlighted previously, studies have shown that reductions in D and D* correlate with tubular edema and decreased extracellular space in renal cold ischemia, while ADC and D have reflected cortical and medullary fibrosis in renal artery stenosis [28]; and in early diabetic nephropathy, D demonstrated superior sensitivity over other IVIM parameters [18]. In our study, CA-induced IRI was acute and severe, as evidenced by structural damage and alterations in IVIM-derived diffusion and perfusion parameters. The observed reductions in IVIM metrics (ADC, D, D*, f) align with prior studies of renal IRI and contrast-induced AKI models, where decreases in diffusion and perfusion parameters closely correlated with tubular epithelial swelling, apoptosis, and histopathological injury [29]. Consistent with previous MRI investigations of IRI, diffusion-related parameters have demonstrated greater sensitivity to microstructural tissue damage, whereas perfusion-related IVIM metrics alone show limited discriminatory power. The selection of D as the primary endpoint is supported both by its well-established biophysical basis and its proven robustness in multiparametric imaging analyses [30, 31]. Collectively, these findings reinforce IVIM MRI as a noninvasive tool to assess renal diffusion and perfusion changes associated with injury, although direct evidence linking IVIM changes to tight junction disruption in CA-induced IRI remains limited.

At the molecular level, our CA model induced significant tubular injury, characterized by epithelial cell swelling and disruption of mitochondrial cristae, consistent with the diffusion changes detected by IVIM MRI. Western blot analysis revealed that, at 24 h post-ROSC, both total Bcl-2 expression and cleaved caspase-3 levels were markedly elevated. These findings suggest that during the early phase of renal injury, both protective and injurious apoptotic pathways are concurrently activated, reflecting a dynamic balance between cell survival and death signals.

Our results are consistent with previous studies. Fu et al reported that AKI following cardiac arrest is commonly associated with increased apoptosis and inflammatory responses, supporting the pathological context of our model [8]. Grünenfelder et al demonstrated that inhibition of caspase-3 promotes Bcl-2 expression and mitigates IRI, highlighting the role of anti-apoptotic signaling as a negative feedback mechanism in apoptosis regulation [32]. Suzuki et al further emphasized that Bcl-2 protects tubular epithelial cells by inhibiting apoptosis, reflecting an intrinsic self-protective response of the kidney under ischemic stress [33]. Moreover, the concurrent upregulation of pro-apoptotic and anti-apoptotic factors in early renal ischemic injury reflects a complex regulatory mechanism under cellular stress: pro-apoptotic proteins such as Bax and caspase-3 initiate apoptosis to eliminate damaged cells, while anti-apoptotic proteins like Bcl-2 delay apoptosis, allowing time for cell survival and repair. The relative balance between these signals likely influences the progression and recovery of renal injury. This dynamic regulatory mechanism provides a theoretical foundation for future therapeutic strategies aimed at modulating apoptotic and protective signaling pathways. Although the simultaneous activation of Bcl-2 and cleaved caspase-3 is biologically plausible and consistent with existing reports, the precise temporal relationship between these signals requires further investigation. Future studies with multiple time points and larger sample sizes are warranted to better elucidate the dynamic interplay between anti-apoptotic and pro-apoptotic signals during renal injury.

Tight junction proteins play a central role in maintaining tubular epithelial integrity. ZO-1 depletion destabilizes intercellular junctions, increasing paracellular permeability and disrupting epithelial polarity [34], whereas occludin loss following IRI compromises tubular structure and promotes epithelial cell shedding [35]. In parallel, kidney injury molecule‑1 is rapidly upregulated in proximal tubular epithelial cells after ischemic insult, serving as a sensitive early biomarker of AKI [36]. In our study, reductions in ZO-1 and occludin, along with decreased aquaporin-3, were indicative of epithelial barrier disruption and impaired tubular water transport. Importantly, these molecular alterations were accompanied by significant declines in IVIM parameters (ADC, D, D*, f), reflecting changes in renal water diffusion and perfusion that were associated with molecular and cellular injury. Diffusion kurtosis imaging (DKI) may further complement IVIM, enhancing sensitivity to early microstructural changes [37–39]. Moreover, the CA-ROSC model more accurately mimics clinical IRI than traditional renal artery clamping, reducing surgical confounders and improving translational relevance [8]. Collectively, these findings support a pathophysiological framework in which tight junction disruption, defective water handling, oxidative stress, and imbalance between tubular injury and repair are reflected in multiparametric MRI metrics, underscoring their potential for early detection of renal injury following CA and resuscitation.

Although urinary and plasma biomarkers have shown potential for early renal injury detection, their clinical utility remains limited due to variability, timing dependence, and lack of standardized thresholds [40, 41]. In this study, we focused on assessing IVIM MRI parameters without evaluating these biomarkers. IVIM MRI offers a noninvasive, multiparametric imaging approach capable of spatially resolving renal microstructure and perfusion without contrast agents, allowing for repeated measurements over time. In the present study, significant changes in multiple IVIM parameters (ADC, D, D*, f, and eADC) were observed between CA and sham groups, suggesting functional alterations in renal water diffusion and microvascular perfusion 24 h after CA. However, the interpretation of these parameters requires baseline or reference values, and considerable overlap was observed between groups for most parameters, highlighting the need for standardized imaging protocols and predefined cut-off values. Building upon the interpretation of IVIM-derived diffusion and perfusion alterations, we integrated these imaging findings with immunohistochemical assessment of aquaporin-3 expression to provide complementary, multi-level insights. Considering that IVIM quantifies water molecule diffusion and perfusion dynamics, while aquaporin-3 functions as a key renal water channel protein, a physiological link between these markers is plausible in modulating renal water homeostasis and microenvironment. This integrative approach may link microstructural imaging abnormalities with molecular changes, potentially enhancing our understanding of the early pathophysiological mechanisms underlying renal IRI post-CA.

Despite these advantages, several limitations should be acknowledged. First, the region-of-interest analysis did not distinguish between the renal cortex and medulla, potentially obscuring region-specific pathophysiological alterations. Future work should incorporate corticomedullary segmentation or voxel-wise analysis to address this spatial heterogeneity. Second, the perfusion-sensitive parameters (D* and f) may be susceptible to motion artifacts. Implementation of respiratory gating, optimized acquisition schemes, and advanced fitting algorithms in future studies could improve the robustness and reliability of these metrics. Third, the assessment of renal function was limited by a constrained urine analysis protocol. A more systematic collection and analysis of urine biomarkers in future investigations would provide a more comprehensive functional evaluation alongside imaging findings. Fourth, while expanding the cohort strengthened the statistical significance of Bcl-2 and cleaved caspase-3 expression—reinforcing evidence of apoptotic regulation—limitations persist. These include the absence of detailed temporal profiling of apoptosis and the need for larger sample sizes to fully delineate its dynamic progression. Longitudinal studies with extended sampling are therefore warranted. Fifth, the mortality rate in the CA group (~33%) is consistent with established rodent models, supporting the biological validity of our study [42]. However, this inherent mortality introduces potential survivorship bias, as animals that survived for terminal analyses may represent a subpopulation with less severe injury. Although the sample size was informed by previous preclinical imaging studies [43, 44] and a *post hoc* power analysis confirmed sufficient power (~0.95) for our primary endpoint (D), these factors remain important caveats. Future investigations with larger cohorts and designs that account for or mitigate survivorship bias are necessary. Sixth, variability in imaging acquisition, processing, and protocols currently limits the broader clinical applicability of IVIM-MRI. Multicenter reproducibility testing and protocol harmonization are essential steps toward clinical translation. Finally, and critically, while IVIM-MRI detected early structural and perfusion alterations indicative of renal IRI, the animals did not meet the standard clinical criteria for AKI (based on Cr, BUN, or urine output) at the 24-h time point. Therefore, our findings should be interpreted as revealing subclinical, imaging-detectable injury that precedes conventional functional markers—a distinction that underscores the potential of IVIM-MRI for early detection but also clarifies the stage of injury captured in this model. Despite these limitations, our study provides statistically robust and biologically meaningful imaging evidence for the early detection of renal IRI following CA.

Future studies integrating IVIM imaging with biomarker assessments and advanced analytic methods are warranted to enhance the sensitivity and clinical applicability of early renal injury detection. Recent studies have advanced the application of multiparametric MRI in renal injury assessment. Notably, a machine learning model utilizing multiparametric MRI data has demonstrated strong predictive capability for pathological grading in a rat model of cold IRI [45]. Additionally, IVIM MRI has been effectively employed to quantitatively evaluate renal perfusion and diffusion alterations in unilateral ureteral obstruction models [46]. These findings support our study by highlighting the increasing value of MRI-based techniques for noninvasive evaluation of kidney damage. They also suggest that combining these imaging methods with machine learning could further improve diagnostic accuracy in future research.

IVIM MRI is a sensitive, noninvasive method for detecting early renal IRI. Changes in its parameters closely reflect tubular cell swelling, microvascular dysfunction, and correspond well with microstructural alterations, histopathology, and molecular markers related to apoptosis and epithelial integrity. However, since these results come from preclinical models, further clinical studies are necessary before IVIM MRI can be considered a reliable tool for early diagnosis in patients. Future work should explore the use of IVIM in other causes of kidney injury, including inflammatory and metabolic conditions, and investigate how machine learning might enhance diagnostic precision. Given the common occurrence of multi-organ dysfunction after CA, simultaneous IVIM assessment of the brain and kidneys could offer valuable insights into systemic IRI. With continued development and validation, this approach may help enable personalized treatment and improve management after resuscitation.

## Supplementary information

**Additional file 1**: **Table S1** Intraclass Correlation Coefficients (ICC) of IVIM MRI Parameters in Right and Left Kidneys of cardiac arrest and sham-operated control Groups. **Table 2** Comparison of renal IVIM-derived parameters between sham and cardiac arrest (CA) groups. **Table 3** Overview of experimental assays and sample sizes used in the cardiac arrest and sham groups. **Table 4** Intraclass correlation coefficients for histopathological and ultrastructural scores in cardiac arrest and sham groups.

## Data Availability

Data will be made available on request.

## Abbreviations

ADC: Apparent diffusion coefficient
AKI: Acute kidney injury
BUN: Blood urea nitrogen
CA: Cardiac arrest
CPR: Cardiopulmonary resuscitation
Cr: Creatinine
D: True diffusion coefficient
D*: Pseudo-diffusion coefficient
eADC: Effective apparent diffusion coefficient
f: Perfusion fraction
IRI: Ischemia–reperfusion injury
IVIM: Intravoxel incoherent motion
MRI: Magnetic resonance imaging
ROI: Region of interest
ROSC: Return of spontaneous circulation
ZO-1: Zonula occludens-1
