# Supplementary material for: Intravoxel incoherent motion MRI for early detection and assessment of renal injury after cardiac arrest and resuscitation in a rat model
Source: Eur Radiol Exp. 2026 May 13;10:63. doi: 10.1186/s41747-026-00729-8 (PMC13172073; doi:10.1186/s41747-026-00729-8)
Supplement: Supplementary file 1 — Additional file 1: Table S1 Intraclass Correlation Coefficients (ICC) of IVIM MRI Parameters in Right and Left Kidneys of cardiac arrest and sham-operated control Groups. Table 2 Comparison of renal IVIM-derived parameters between sham and cardiac arrest (CA) groups. Table 3 Overview of experimental assays and sample sizes used in the cardiac arrest and sham groups. Table 4 Intraclass correlation coefficients for histopathological and ultrastructural scores in cardiac arrest and sham groups. [file 41747_2026_729_MOESM1_ESM.pdf]

Intravoxel incoherent motion MRI for early detection and assessment of renal injury after cardiac arrest and resuscitation in a rat model

ELECTRONIC SUPPLEMENTARY MATERIAL

Supplemental methods

Randomization and group allocation

A total of 24 male SD rats were randomly assigned to either the CA group (n = 15) or the sham-operated (sham) group (n = 9) using a computer-generated randomization sequence. After the experimental procedure, surviving animals underwent MRI acquisition under anesthesia at 24 hours. Animals were then euthanized, blood samples were collected from the inferior vena cava, and both kidneys were harvested for subsequent histological and molecular analyses [1].

Cardiac arrest model and experimental procedures

All rats were fasted for 8–10 hours prior to the procedure. Anesthesia was induced via intraperitoneal injection of sodium pentobarbital (45 mg/kg). A 14G endotracheal catheter (Abbocath-T, USA) was inserted to allow mechanical ventilation. Simultaneously, a 23G polyethylene catheter (Abbocath-T, USA) was inserted into the left femoral artery for continuous monitoring of mean arterial pressure (MAP). Hemodynamic parameters were recorded in real time using the WinDaq data acquisition system (USA).

CA was induced by clamping the endotracheal tube to cause asphyxia and was defined as a sustained MAP ≤ 30 mmHg. After 5 minutes of untreated CA, CPR was initiated, consisting of mechanical ventilation and manual chest compressions. Two minutes after CPR initiation, epinephrine (0.01 mg/kg) was administered via the femoral artery and repeated every 2 minutes as needed. In cases of ventricular fibrillation, up to three biphasic electrical shocks (2 J each) were delivered. ROSC was defined as MAP ≥ 60 mmHg along with restoration of a sustained supraventricular rhythm for at least 5 minutes. After ROSC, all catheters and monitoring devices were removed, and animals were allowed to recover for the subsequent 24 hours. Resuscitation efforts were terminated if ROSC was not achieved after 6 minutes of continuous CPR [2].

Sham-operated rats underwent identical anesthesia, intubation, and femoral artery catheterization without CA induction. At 24 hours post-procedure, all animals underwent MRI scanning under anesthesia. While still anesthetized, blood samples were collected from the inferior vena cava, and both kidneys were harvested. Physiological variables—including MAP, heart rate (HR), respiratory rate (RR), oxygen saturation (SpO_2), body temperature, and anesthesia duration—were recorded at baseline (pre-CA) and again 24 hours after cardiopulmonary resuscitation (post-CPR). Animals were then humanely euthanized in accordance with institutional guidelines.

Ethical approval and animal welfare

All experimental procedures were conducted in accordance with institutional guidelines for the care and use of laboratory animals and were approved by the Institutional Animal Care and Use Committee (IACUC). Investigators responsible for MRI acquisition and data analysis were blinded to group allocation. Given the acute nature of the study design and the predefined endpoint at 24 hours after ROSC, animals were maintained under deep anesthesia throughout MRI scanning and subsequent tissue collection. Baseline vital signs, including body temperature, heart rate, and respiratory rate, were recorded prior to MRI scanning, and these parameters were reassessed within 24 hours post-scanning to ensure adequate anesthetic depth and minimize procedural stress. Due to limitations imposed by the MRI environment, continuous real-time monitoring of body temperature during scanning was not feasible; however, clinical observations and pre- and post-scan measurements were utilized to ensure animal welfare. Because animals did not regain consciousness prior to euthanasia, postoperative analgesia was not administered, but animals were closely monitored for any signs of distress. Humane euthanasia was performed immediately after tissue collection, with all procedures designed to minimize animal suffering.

MRI acquisition

Rats were scanned by 3.0 T superconducting MRI (Prisma, Siemens), using an 80 mT/m maximum gradient strength and a 200 T/m/s slew rate. An 8-channel rodent-specific coil (Mouse Coil-M, CL33000001) was used for signal reception. Imaging was performed under free-breathing conditions using a multi–b-value single-shot echo-planar imaging (EPI) sequence with integrated shimming (iShim) in the coronal plane. Imaging parameters were as follows: TR/TE = 2300/74 ms; field of view = 140 × 114 mm^2; slice thickness = 3.0 mm; acquisition matrix = 120 × 98; and reconstructed voxel size = 0.6 × 0.6 × 3.0 mm^3. Diffusion weighting employed ten b-values (0, 10, 20, 30, 50, 100, 200, 300, 500, and 800 s/mm^2) applied along three orthogonal directions. The total acquisition time was 7 minutes and 9 seconds. The mean signal-to-noise ratio (SNR) was 20.87, ensuring adequate image quality for robust IVIM parameter estimation.

IVIM data processing and analysis were performed using the IMAgenGINE software platform (WWW.VUSION.COM.CN) [3]. A two-step fitting strategy was adopted. High b-value signals (≥200 s/mm^2) were first isolated to estimate the D. With D fixed, all b-values were subsequently fitted to a bi-exponential IVIM model to derive the f, D*, ADC, and eADC.

Raw images were first imported into the IMAgenGINE platform for automatic motion correction and parametric map generation. However, region of interest (ROI) delineation of the renal parenchyma was performed manually. Specifically, the renal outer boundary was initially defined on T2-weighted images, with exclusion of the renal sinus, collecting system, and large vessels based on characteristic signal intensities to ensure accurate segmentation. Two experienced observers, fully blinded to the group allocation (CA or sham), independently drew the whole-kidney ROIs along the outer contour of the renal parenchyma. This manual delineation approach was chosen to maximize accuracy and reproducibility, while minimizing potential bias.

Given the indistinct renal contours and blurred corticomedullary differentiation during the early post-resuscitation period, which may compromise reliable segmentation of cortex and medulla, whole-kidney ROIs were employed to improve reproducibility and consistency. ROIs were independently delineated for the left and right kidneys, with IVIM parameters extracted separately and then averaged to obtain a single observation per animal. This approach was used to avoid the non-independence (clustered structure) of bilateral kidney data and to ensure statistical inference was conducted at the animal level.

IVIM-derived quantitative parameters—including *ADC*, *D*, *D**, perfusion fraction(f) and effective apparent diffusion coefficient(eADC)—were extracted from the renal regions and subjected to further statistical analysis. Quantitative diffusion analysis was performed using IVIM MRI. The biexponential IVIM model was fitted to the diffusion-weighted signals according to the following equation:

$$S/S_0 = \exp(-b \cdot eADC)$$

where S represents the signal intensity at a given b-value, S_0 is the signal at b = 0, and eADC denotes the exponential apparent diffusion coefficient. The eADC parameter reflects overall diffusion attenuation across all b-values and provides a composite measure of tissue diffusivity, which has been shown to correlate with renal microstructural alterations and IRI [4].

The ADC was calculated by fitting a monoexponential decay model to the diffusion-weighted signal intensities acquired at multiple b-values within each ROI. The model follows the equation:

$$S(b)=S_0 \times e^{-b \times ADC}$$

where S(b)represents the signal intensity at a given b-value b, and $S_0$is the signal intensity at b=0 s/mm². Within each ROI, signal intensities were logarithmically transformed, and linear regression of lnS(b) against the b-values was performed. The negative slope of this regression line was defined as the ADC. The eADC differs from ADC in that it is derived from signal decay over a selected subset of b-values or through alternative fitting approaches designed to emphasize specific diffusion or perfusion components. While ADC reflects the combined effects of molecular diffusion and microcirculation-related pseudo-diffusion, eADC can provide complementary information by highlighting the exponential signal attenuation characteristics within restricted b-value ranges.

The biexponential IVIM model was fitted to the diffusion-weighted signals according to the following equation:

$$S/S_0 = f \cdot \exp(-b \cdot D^*) + (1 - f) \cdot \exp(-b \cdot D)$$

Based on the biophysical interpretation of IVIM parameters and consensus from previous IRI studies, the D within the whole-kidney ROI was predefined as the primary imaging endpoint. IVIM-derived D was defined as the primary imaging endpoint. f represents the contribution of microvascular perfusion to the diffusion-weighted signal (range, 0–1). D corresponds to the slow diffusion component of water molecular motion (mm²/s), and D* reflects the fast diffusion component associated with incoherent microcirculatory motion (mm²/s). All quantitative parameters were derived from the standard IVIM biexponential model.

Renal function assessment

Blood was drawn into syringes preloaded with 3.8% sodium citrate. Plasma was obtained by centrifugation at 4 °C for 10 min at 4,000 × g. Plasma Cr and BUN were measured using an i-STAT 1 handheld analyzer (Model 300-G) with CHEM8+ cartridges (Abbott Point of Care Inc., USA), according to the manufacturer's instructions. CHEM8+ cartridges provide rapid point-of-care measurements traceable to reference standards and are commonly applied for assessment of renal function in preclinical and clinical samples. Each sample was measured in triplicate and each assay independently repeated three times to ensure analytical reliability. For subsequent statistical analysis, changes in Cr and BUN (ΔCr and ΔBUN) were calculated as post-procedure values minus pre-procedure values to reflect the magnitude and direction of renal functional change.

Histopathology

After MRI, rats were euthanized, and kidneys were collected for histological analysis. Kidney tissues from animals selected for histopathological analysis cut into approximately 3–5 mm thick slices and fixed in 10% neutral-buffered formalin (Sigma-Aldrich, St. Louis, MO, USA) for 24 h. Fixed tissues were then dehydrated through a graded ethanol series, embedded in paraffin (Leica, Wetzlar, Germany), and sectioned at 5 μm thickness. Morphological assessment was performed using hematoxylin and eosin (H&E) staining (Sigma-Aldrich, St. Louis, MO, USA).

Histopathological scoring was independently performed by two pathologists blinded to group assignments to ensure objectivity and minimize bias. Renal injury was semi-quantitatively assessed on H&E-stained sections using a 4-point scale (0–3), focusing on apoptotic features and cellular alterations. A score of 0 indicated normal renal morphology without apoptotic bodies or cellular damage; 1 represented mild presence of apoptotic bodies, slight nuclear pyknosis, and mild cytoplasmic eosinophilia; 2 corresponded to moderate apoptotic bodies accompanied by pronounced nuclear pyknosis and fragmentation, as well as moderate cytoplasmic changes; and 3 reflected severe apoptosis and necrosis, with extensive nuclear fragmentation and marked cytoplasmic degeneration.

Histopathological analysis included five animals per group. For each animal, five kidney sections were evaluated, and the scores from these sections were averaged to yield a single mean histopathological score reflecting the overall renal injury in that animal.

Transmission electron microscopy (TEM)

Kidney samples were fixed overnight at 4°C in 2.5% glutaraldehyde (Electron Microscopy Sciences, Hatfield, PA, USA) in 0.1 M phosphate buffer (pH 7.4), followed by post-fixation with 1% osmium tetroxide (Electron Microscopy Sciences, Hatfield, PA, USA) for 1 h. Samples were dehydrated through a graded ethanol series and embedded in Epon resin (TAAB, Berkshire, UK). Ultrathin sections (~100 nm) were prepared using an ultramicrotome (Leica EM UC7, Wetzlar, Germany) and examined under a transmission electron microscope (Tecnai G2, FEI, Hillsboro, OR, USA) at 13,500× magnification. Mitochondrial structure and cross-sectional area were quantified using ImageJ software (NIH, Bethesda, MD, USA). Ultrastructural analysis using electron microscopy was independently conducted by two observers blinded to the experimental groups to ensure objective and unbiased evaluation. Ultrastructural injury was assessed by TEM using a comparable 4-point scoring system focusing on mitochondrial morphology, nuclear membrane integrity, and chromatin condensation. A score of 0 reflected normal ultrastructure without abnormalities; 1 indicated mild mitochondrial alterations, such as slight cristae disruption and subtle nuclear membrane wrinkling; 2 corresponded to moderate mitochondrial swelling or shrinkage, evident cristae disruption or loss, nuclear membrane damage, and chromatin condensation; and 3 represented severe mitochondrial damage characterized by cristae loss or fragmentation, profound nuclear membrane disruption, and chromatin fragmentation or extreme condensation. The ultrastructural scoring was performed on sections from five independent animals per group. For each animal, three non-overlapping fields were randomly selected and examined to provide a representative evaluation.

Detection of Renal Injury and Tight Junction Proteins by Immunohistochemistry and Immunofluorescence

Rat kidneys were fixed in 4% paraformaldehyde (Servicebio, China) for 12–24 h, embedded in paraffin, and sectioned at 4 μm. Prior to antigen retrieval using a pressure cooker, the paraffin sections were deparaffinized in xylene and rehydrated through a graded ethanol series. Heat-mediated antigen retrieval was performed using Tris-EDTA buffer (pH 9.0, Servicebio, China) in a pressure cooker for 2 min. For immunohistochemistry (IHC), sections were incubated overnight at 4 °C with primary rabbit antibodies against occludin and ZO-1 (1:1000, Servicebio, China), followed by HRP-conjugated goat anti-rabbit IgG (1:200, Servicebio, China) at room temperature for 1 h, and visualized using 3,3′-diaminobenzidine (DAB). For immunofluorescence (IF), sections were incubated overnight at 4 °C with rabbit anti-KIM-1 (1:200, Proteintech, China), followed by CoraLite®488-conjugated goat anti-rabbit IgG (1:200, Proteintech, China) for 1 h at room temperature, with nuclei counterstained using DAPI (Sigma-Aldrich, St. Louis, MO, USA). Five non-overlapping images per animal were randomly captured from the renal cortex (five animals per group). Immunohistochemical (IHC) and immunofluorescence (IF) staining analyses were performed on renal cortex tissues from five animals per group. For each animal, five non-overlapping cortical fields were randomly selected under consistent imaging settings. IHC staining was quantified by average optical density (AOD) using Image-Pro Plus 6.0 (Media Cybernetics, Rockville, MD, USA), while IF signals were measured as corrected mean fluorescence intensity (MFI) after background subtraction using ImageJ software. To minimize subjective bias, two blinded observers independently reviewed all images, resolving discrepancies by consensus. The mean value from the five fields per animal was used for statistical analysis, with the animal considered the statistical unit to avoid pseudo-replication.

Western Blotting

Kidney tissues collected 24 h after resuscitation were homogenized on ice in RIPA lysis buffer containing protease and phosphatase inhibitors (Beyotime, Shanghai, China). Lysates were centrifuged at 12,000 × g for 15 min at 4 °C, and supernatants were collected for protein quantification using a bicinchoninic acid (BCA) assay (Thermo Fisher Scientific, Waltham, MA, USA). Equal amounts of protein (30–50 μg) were separated by 10% SDS-polyacrylamide gels and transferred to polyvinylidene difluoride (PVDF) membranes (Millipore, Burlington, MA, USA). Membranes were blocked with 5% non-fat milk in Tris-buffered saline with 0.1% Tween-20 (TBST) for 1 h at room temperature and then incubated overnight at 4 °C with primary antibodies against Bcl-2 (rabbit, 1:1,000; Zen Bioscience, Guangzhou, China), cleaved Caspase-3 (rabbit, 1:1,000, Zen Bioscience), AQP3 (rabbit, 1:500, Zen Bioscience), and β-actin (rabbit, 1:5,000; Abcam, Cambridge, UK) as a loading control. After three washes in TBST, membranes were incubated with horseradish peroxidase (HRP)-conjugated anti-rabbit secondary antibodies (1:5,000; Abcam) for 1 h at room temperature. Protein bands were detected using enhanced chemiluminescence reagents (Thermo Fisher Scientific) and imaged with a ChemiDoc MP system (Bio-Rad, Hercules, CA, USA). Western blot analyses were performed on kidney tissue samples obtained from all animals that successfully completed the MRI examinations (CA group, n = 10; sham group, n = 9). Protein extraction followed standard protocols. Band intensities were quantified using ImageJ software (NIH, Bethesda, MD, USA) and normalized to β-actin as a loading control. Each biological sample was measured once.

Statistical Analysis

Statistical analyses were conducted using GraphPad Prism version 10.0 (GraphPad Software, San Diego, CA, USA). The individual animal was used as the unit of analysis. Data distribution was assessed by the Shapiro–Wilk test. Normally distributed variables are presented as mean ± standard deviation (SD), while non-normally distributed variables are expressed as median with interquartile range (IQR). Between-group comparisons were performed using independent-samples t-tests for normally distributed data or Mann–Whitney U tests for non-normally distributed data. The diffusion coefficient D was prespecified as the primary imaging endpoint and used for group comparisons. All statistical tests were two-tailed, with p-values less than 0.05 considered statistically significant. Exact p-values are reported to three decimal places, with values below 0.001 reported as $p < 0.001$. A priori power analysis based on preliminary data and relevant literature confirmed that the sample size provided sufficient statistical power. Given that multiple IVIM parameters (D, D*, f, ADC, eADC) were analyzed, the Benjamini-Hochberg procedure was applied to control the false discovery rate (FDR) at 0.05 across all hypothesis tests involving these parameters, thereby mitigating the risk of false positives arising from multiple comparisons.

Reference

1. Magnet IAM, Ettl F, Schober A et al (2017) Extracorporeal Life Support Increases Survival After Prolonged Ventricular Fibrillation Cardiac Arrest in the Rat. Shock 48:674–680. https://10.1097/SHK.0000000000000909
2. Wang P, Li Y, Yang Z et al (2018) Inhibition of dynamin-related protein 1 has neuroprotective effect comparable with therapeutic hypothermia in a rat model of cardiac arrest. Transl Res 194:68–78. https://10.1016/j.trsl.2018.01.002
3. Yang M, Yan Y, Wang H (2019) IMAge/enGINE: a freely available software for rapid computation of high-dimensional quantification. Quant Imaging Med Surg 9:210–218. https://10.21037/qims.2018.12.03
4. Fu Z-Y, Wu Z-J, Zheng J-H et al (2019) The incidence of acute kidney injury following cardiac arrest and cardiopulmonary resuscitation in a rat model. Ren Fail 41:278–283. https://10.1080/0886022X.2019.1596819

Supplemental Materials

Table 1 Intraclass Correlation Coefficients (ICC) of IVIM MRI Parameters in Right and Left Kidneys of cardiac arrest and sham-operated control Groups

Parameter	ICC CA Group Right Kidney	95 %CI	P	ICC CA Group Left Kidney	95 %CI	P	ICC SHAM Group Right Kidney	95 %CI	P	ICC SHAM Group Left Kidney	95 %CI	P
D	0.987	0.96 – 0.997	<0.0001	0.958	0.87 – 0.99	<0.0001	0.987	0.96 – 0.997	<0.0001	0.964	0.88 – 0.991	<0.0001
D*	0.984	0.95 – 0.996	<0.0001	0.972	0.91 – 0.994	<0.0001	0.974	0.93 – 0.993	<0.0001	0.974	0.93 – 0.993	<0.0001
ADC	0.831	0.49 – 0.96	0.0012	0.973	0.92 – 0.994	<0.0001	0.978	0.94 – 0.994	<0.0001	0.991	0.97 – 0.997	<0.0001

Parameter	ICC CA Group Right Kidney	95 %CI	P	ICC CA Group Left Kidney	95 %CI	P	ICC SHAM Group Right Kidney	95 %CI	P	ICC SHAM Group Left Kidney	95 %CI	P
f	0.952	0.85 – 0.99	< 0.0001	0.952	0.85 – 0.99	<0.0001	0.992	0.98 – 0.998	<0.001	0.984	0.95 – 0.996	<0.001
eADC	0.961	0.88 – 0.99	< 0.0001	0.954	0.86 – 0.99	<0.0001	0.892	0.64 – 0.97	0.0003	0.954	0.86 – 0.99	<0.001

Intraclass correlation coefficients represent the test–retest reliability of IVIM MRI parameters measured in the right and left kidneys of the cardiac arrest and sham-operated control groups. Higher ICC values indicate better reproducibility and measurement reliability. *ADC* Apparent diffusion coefficient, *CA* Cardiac arrest, *CI* Confidence interval, *D* True diffusion coefficient, *D** Pseudodiffusion coefficient, *eADC* Effective apparent diffusion coefficient, *f* Perfusion fraction; *ICC* Intraclass correlation coefficient, *MRI* Magnetic resonance imaging, *SHAM* Sham-operated control.

Table 2 Comparison of renal IVIM-derived parameters between sham and cardiac arrest (CA) groups

Parameter	Sham group (n = 9)	CA group (n = 10)	P value
true diffusion coefficient (D)	3189 ± 711	2273 ± 275	0.002
pseudo-diffusion coefficient (D*)	13085± 3834	8843 ± 3552	0.023
Apparent diffusion coefficient (ADC)	3460± 453	2462 ± 259	<0.001
perfusion fraction (f)	0.38 ± 0.07	0.19 ± 0.04	<0.001
effective ADC (eADC)	0.47 ± 0.08	0.35 ± 0.05	0.001

Data are expressed as mean ± SD. For each animal, IVIM-derived parameters were calculated by averaging the values obtained from the left and right kidneys. Between-group comparisons (sham vs cardiac arrest) were performed using an independent-samples *t* test for normally distributed data or the Mann–Whitney *U* test for non-normally distributed variables, as appropriate. Whole-kidney regions of interest (ROIs) were manually delineated along the outer contour of the renal parenchyma, without separation of the cortex and medulla. *ROIs* Regions of interest, *SD* standard deviation

Table 3 Overview of experimental assays and sample sizes used in the cardiac arrest and sham groups

Assay	Cardiac Arrest group (n)	Sham group (n)
IVIM MRI	10	9
Morphological analysis (LM + TEM)	5	5
Immunohistochemistry	5	5
Immunofluorescence	5	5
Western blot	10	9

The number of animals analyzed in each experimental assay is shown for the cardiac arrest and sham groups. Whole-kidney regions of interest (ROI) were used for IVIM MRI analysis. Histopathological evaluation employed semi-quantitative scoring of hematoxylin and eosin stained sections. Electron microscopy assessed representative tissue sections. Immunohistochemistry and immunofluorescence were performed for protein localization and expression analyses, respectively. Western blot experiments included independent biological replicates. *IVIM* Intravoxel incoherent motion, *MRI* Magnetic resonance imaging, *ROI* Regions of interest

Table 4 Intraclass correlation coefficients for histopathological and ultrastructural scores in cardiac arrest and sham groups.

Group	Parameter	intraclass correlation coefficient	95 % confidence interval	P value
cardiac arrest	Histopathological Score	0.845	0.57 – 0.96	0.0006
cardiac arrest	Ultrastructural Score	0.810	0.45 – 0.95	0.0012
sham-operated control	Histopathological Score	0.780	0.40 – 0.94	0.0023
sham-operated control	Ultrastructural Score	0.900	0.70 – 0.98	0.0002

Intraclass correlation coefficients with 95% confidence intervals and corresponding p-values are presented for histopathological and ultrastructural scores in the cardiac arrest and sham-operated control groups, reflecting the reliability of scoring assessments.
